# Origin of Hypofunctional CD103^+^ NK Cells in Cirrhosis‐Associated Ascites

**DOI:** 10.1002/eji.202451311

**Published:** 2025-06-11

**Authors:** Christian Niehaus, Daniel Geanon, Ayesha Lietzau, Marija Jankovic, Christopher Maucourant, Benjamin Maasoumy, Ernesto Sparrelid, Heiner Wedemeyer, Julia Kahlhöfer, Christine S. Falk, Itzel Medina Andrade, Andrea Ponzetta, Niklas K. Björkström, Anke R.M. Kraft, Markus Cornberg, Benedikt Strunz

**Affiliations:** ^1^ Department of Gastroenterology, Hepatology, Infectious Diseases and Endocrinology Hannover Medical School Hannover Germany; ^2^ Centre for Individualised Infection Medicine (CiiM) a joint venture between the Helmholtz Centre for Infection Research (HZI) and Hannover Medical School (MHH) Hannover Germany; ^3^ Twincore, Centre for Experimental and Clinical Infection Research a joint venture between the Helmholtz Centre for Infection Research (HZI) and the Hannover Medical School Hannover Germany; ^4^ Center for Infectious Medicine Department of Medicine Huddinge Karolinska Institutet Karolinska University Hospital Stockholm Sweden; ^5^ German Center for Infection Research (DZIF) Partner‐site Hannover‐Braunschweig Hannover Germany; ^6^ Division of Surgery and Oncology Department of Clinical Science Intervention and Technology Karolinska Institutet Karolinska University Hospital Stockholm Sweden; ^7^ Cluster of Excellence RESIST (EXC 2155) Hannover Medical School Hannover Germany; ^8^ German Center for Infection Research HepNet Study‐House German Liver Foundation Hannover Germany; ^9^ Institute of Transplant Immunology Hannover Medical School Hannover Germany

**Keywords:** gut leakage, liver cirrhosis, natural killer cells, peritoneal cavity

## Abstract

The occurrence of ascites is a frequent complication associated with the decompensation of liver cirrhosis. While it is known that cirrhosis leads to altered immune responses in the periphery, the immunological milieu of ascites remains poorly understood. In this study, we investigate the role and origin of natural killer (NK) cells in cirrhosis‐associated ascites. Using high‐dimensional flow cytometry and cytokine analysis, we analyzed matched peripheral blood and ascites fluid alongside liver and duodenum samples to discern tissue‐specific differences. Interestingly, a subset of peritoneal NK cells displayed high expression of the tissue‐residency receptor CD103. This subset of CD103^+^ ascites NK cells was distinct from blood, liver, and intestinal NK cells and presented with a less activated phenotype coupled with reduced effector capacity. Investigating their origin, we could identify that cytokines present in ascites, here predominantly IL‐15 in synergy with IL‐21 and TGFβ, can induce CD103 expression and that ascites supernatant further facilitates this process. These results indicate that the ascites in patients with decompensated liver cirrhosis harbor a heterogenous subset of CD103^+^ NK cells that is likely induced by the cytokine milieu.

## Graphical abstract

Ascites is one hallmark of decompensated liver cirrhosis. NK cells in cirrhosis‐associated ascites are a heterogeneous group of cells with altered functionality and CD103 expression identifies a subpopulation that is likely to be induced by cytokines present in the ascites fluid.

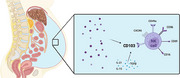

**Abbreviations**
ADCC: antibody‐dependent cellular cytotoxicity
ARC: alcohol‐related liver cirrhosis
IFNγ: interferon γ
lr: liver‐resident
MNC: mononuclear cells
NK cells: natural killer cells
PBMC: peripheral blood mononuclear cells
SBP: spontaneous bacterial peritonitis
TNF: tumor necrosis factor
UMAP: Uniform Manifold Approximation and Projection

## 1 Introduction

Dysregulated immune responses are a known feature of liver cirrhosis [1, 2]. This so‐called cirrhosis‐associated immunodeficiency syndrome is believed to be a major cause of elevated infection rates observed in patients with cirrhosis, subsequently leading to an overall 1‐year cumulative mortality of approximately 50% [3, 4, 5, 6]. A frequent focus of such infection in decompensated cirrhosis is, among others, the peritoneal cavity [7]. Therefore, it is important to investigate the immune milieu in ascites in more detail to understand why patients with liver cirrhosis are at such high risk for ascitic infections. Indeed, a recent single‐cell atlas effort, covering both mice and humans, revealed considerable heterogeneity and species differences among peritoneal myeloid cells [8], but less is known regarding lymphocytes.

A lymphocyte subset that has previously been shown to be important for immunity in acute and chronic viral infections and anticancer immune responses are natural killer (NK) cells [9, 10]. More specifically, NK cells have been shown to be essential for the control of viral hepatitis [11, 12]. Accumulating evidence suggests that NK cells not only play an important role in infection control but also contribute to the pathogenesis of liver injury and inflammation [13]. NK cells are not only found in blood but also in organs and at barrier sites where they participate in host defense and tissue function [14, 15, 16, 17]. Of interest, their phenotype and function are organ‐ and niche‐dependent [17, 18, 19, 20]. For example, liver‐resident (lr) NK cells have been shown to express CXCR6 but lack CD16 [21, 22]. On the other hand, the expression of CD103 and CD49a is found on NK cell subsets located intraepithelial, such as in the intestine, lung, or uterus [18, 23–26]. It was recently shown that NK cells are present in the ascites and that they contribute to the antibacterial immune response [27]. Yet, the composition and origin of ascites‐resident NK cells and their global functionality remain to be elucidated.

In this study, we performed high‐dimensional flow cytometry on ascites samples of cirrhosis patients and related the results to those obtained from peripheral blood, liver, and duodenum analysis. We also investigated the cytokine milieu in ascites to obtain a comprehensive picture of peritoneal NK cells and the environment they reside. We demonstrate that peritoneal NK cells in cirrhotic patients are a heterogeneous population with reduced effector capacity and that CD103 marks a subpopulation of hypofunctional NK cells that may be induced by the cytokine milieu present in the ascites.

## 2 Results

### 2.1 NK Cells Are Abundant in Patients with Decompensated Liver Cirrhosis and Further Enriched in the Peritoneal Cavity

**FIGURE 1:**
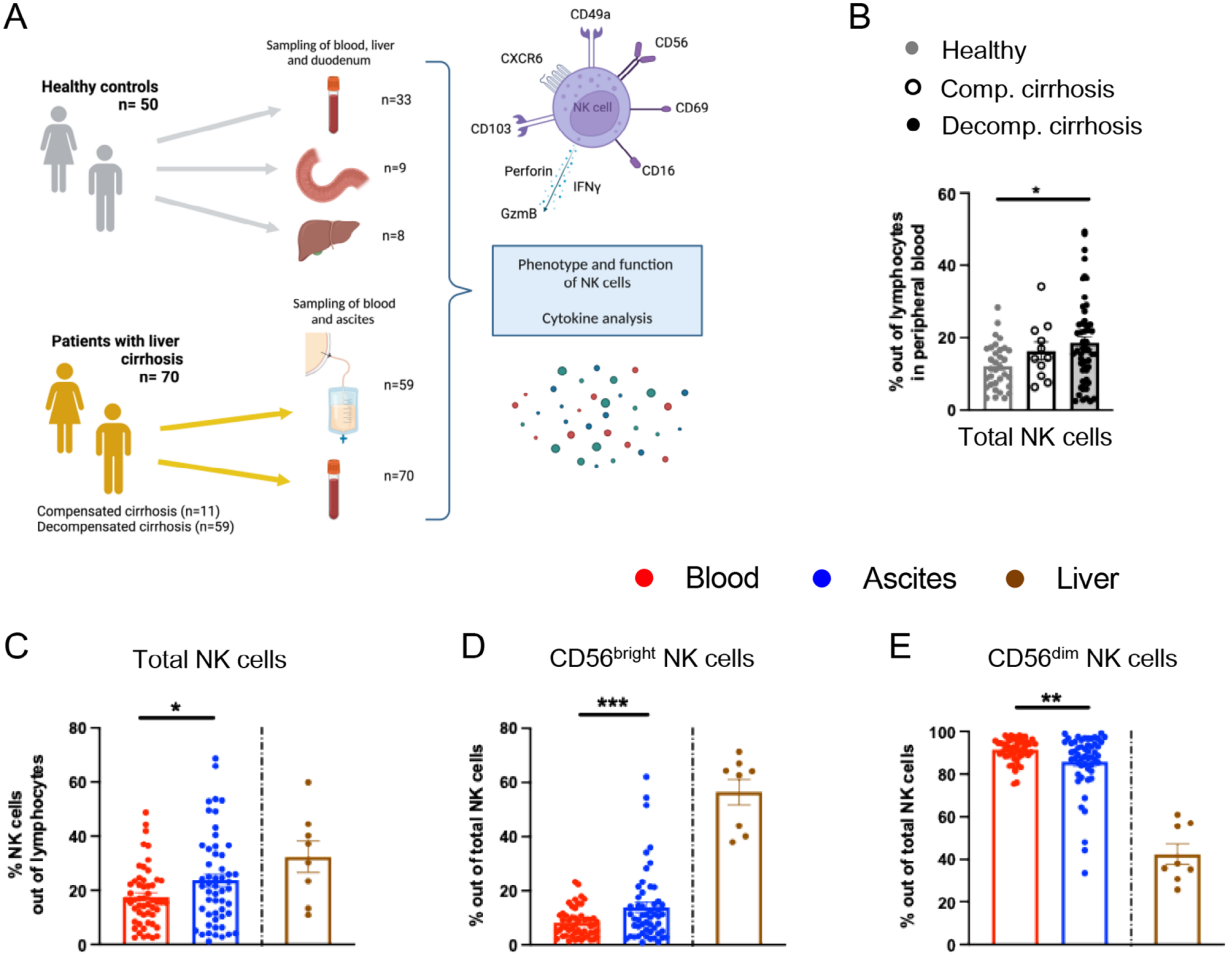
Study outline and frequency of NK cells in patients with liver cirrhosis. (A) Study outline and workflow. (B) Frequency of total NK cells out of lymphocytes in the peripheral blood of patients with decompensated liver cirrhosis (*n* = 53) compared with patients with compensated liver cirrhosis (*n* = 11), and healthy controls (*n* = 33). (C) Frequency of total NK cells out of lymphocytes in blood compared with matched ascites samples (*n* = 53), and liver samples from unrelated controls (*n* = 8) as a reference. (D, E) Frequency of (D) CD56^bright^ and (E) CD56^dim^ NK cells out of total NK cells in the ascites compared with matched blood samples of patients with decompensated liver cirrhosis (*n* = 53), as well as liver samples (*n* = 8) displayed as a reference. Kruskal Wallis test was performed for multiple comparisons. A paired *t*‐test was used to identify significance for normally distributed values, whereas the Wilcoxon test was performed on nonparametric datasets. Liver samples were run in a separate experiment and displayed as a reference without performing statistical tests. *p < 0.05; **p < 0.01; ***p < 0.001. Schematic figure created with BioRender.com (Strunz (2025) https://BioRender.com/7qi2sp6).

In this study, we sought to estimate the origin and the role of NK cells in the immune compartment ascites (Figure 1A). To this end, 59 patients with decompensated and 11 patients with compensated liver cirrhosis were sampled, as well as healthy controls and tissue samples from the liver and duodenum (Figure 1A). First, we compared the frequency of total NK cells in ascites and/or peripheral blood in patients with compensated and decompensated liver cirrhosis as well as peripheral blood from healthy controls and liver samples via multicolor flow cytometry (gating strategy is shown in Figure S1A). NK cell frequency was significantly increased in the blood of patients with decompensated liver cirrhosis compared with healthy controls (Figure 1B). Of interest, when comparing matched blood and ascites samples from patients with decompensated liver cirrhosis, NK cells were further enriched in the peritoneal cavity compared with peripheral blood (Figure 1C). In addition, we noted a shift in the NK cell compartment toward a higher proportion of CD56^bright^ NK cells in ascites (Figure 1D,E). As expected, liver tissue contained a larger fraction of CD56^bright^ NK cells (Figure 1D,E). Thus, NK cells are increased in patients with liver cirrhosis compared with healthy controls and are further enriched in ascites upon decompensation.

### 2.2 Specific Tissue‐Residency Marker Imprint on Ascites NK Cells

**FIGURE 2:**
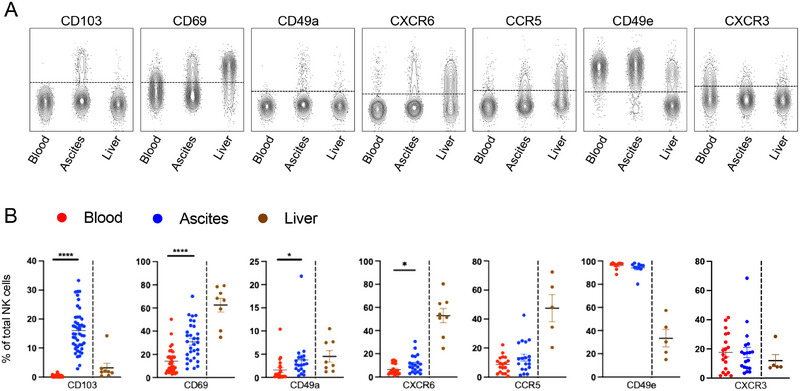
Specific tissue‐residency marker imprint on ascites NK cells. (A) Representative concatenated FACS plots displaying tissue‐residency marker expression on NK cells from blood, ascites, and liver samples. (B) Frequencies of the indicated tissue‐residency markers on total NK cells in matched blood and ascites (*n* = 10–45) from patients with decompensated liver cirrhosis, as well as unmatched liver (*n* = 5–8) samples from unaffected liver tissue acquired during liver resection. For comparison of paired blood and ascites samples, the Wilcoxon test was performed on nonparametric datasets. Liver samples are displayed as a reference and were run in a separate experiment. *p < 0.05; **p < 0.01; ***p < 0.001; ****p < 0.0001.

It is known that tissue‐resident lymphocytes can adapt to the local environment [17, 24, 28, 29]. In that context, CD69, CD103, and CD49a are of importance for the retention of lymphocytes in the different compartments and CXCR6 has been shown to be a marker for lrNK cells [24, 25, 28, 30, 31]. Given the elevated frequency of NK cells in ascites compared with peripheral blood, we, therefore, studied the tissue‐residency profile of peritoneal NK cells in more detail and compared it with that of matched blood, using unrelated liver tissue as a reference. Expression of CD103 was nearly exclusively observed on NK cells from ascites while being nearly absent in the liver and blood (Figure 2A,B). Other tissue‐residency markers, such as CD69 and CD49a, were elevated both in ascites and liver compared with peripheral blood (Figure 2A,B). Distinct patterns were also found for CXCR6 and CCR5, both of which were rarely expressed on peritoneal NK cells but frequently expressed on liver NK cells. In contrast, integrin CD49e was frequently found on ascites and blood NK cells but not on liver NK cells (Figure 2A,B). No relevant differences in expression of CXCR3 were observed (Figure 2A,B). Of note, the chemokine receptors CXCR1 and CCR2 were less expressed on ascites NK cells compared with blood, whereas CXCR4 was upregulated on ascites NK cells (Figure S1B).

In conclusion, ascites‐derived NK cells exhibit a distinct tissue‐residency marker profile marked by high expression of CD103.

### 2.3 High‐Dimensional Data Analysis Reveals Distinct Clusters in Blood, Ascites, and Liver

**FIGURE 3:**
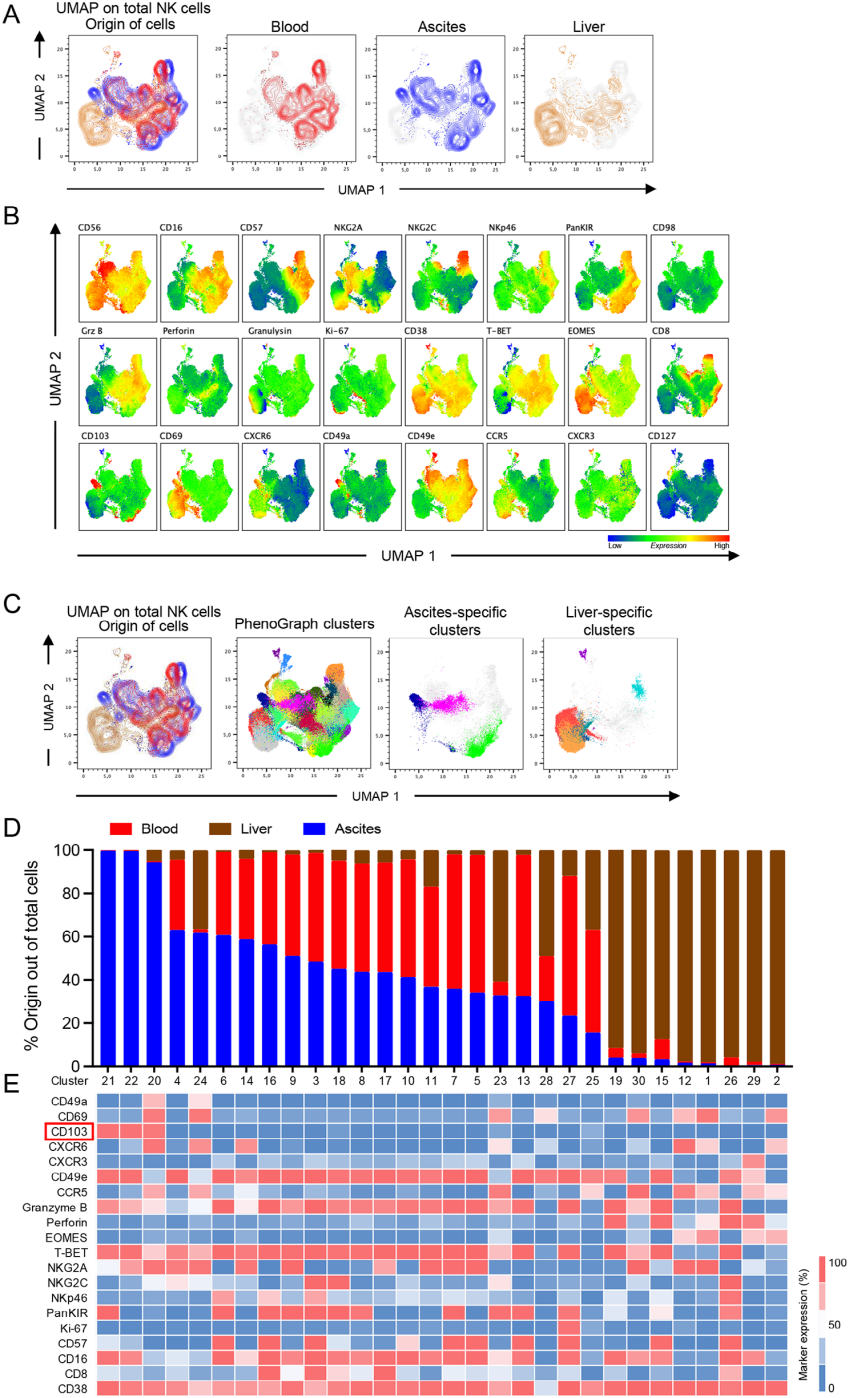
High‐dimensional data analysis reveals distinct clusters in blood, ascites, and liver. (A, B) UMAP plots of total NK cells displaying the three different origins (blood, ascites, liver) of total NK cells, analyzed were five matched ascites and blood samples from patients with decompensated liver cirrhosis as well as five unmatched liver samples with 20.000 NK cells exported from each sample. (B) Expression levels of the indicated markers in the UMAP analysis. (C) Phenograph analysis identifying 30 different clusters within NK cells from blood, ascites, and liver. (D, E) Relative abundance of each of the identified Phenograph clusters in blood, ascites, and liver (D), and the respective phenotype (E).

Given the increased expression of CD103 on ascites NK cells, we next studied if this reflected a general expression of NK cells in the ascites or if it identified a distinct subset. Therefore, we applied Uniform Manifold Approximation and Projection (UMAP) analysis followed by Phenograph clustering on NK cells from peripheral blood, matched ascites, and liver tissue from unrelated controls (Figure 3A–E). Of note, NK cells from peripheral blood and ascites displayed a significant overlap which is reflected in a large fraction of CD56^dim^ NK cells with high expression of T‐bet, CD57, Granzyme B, and/or panKIR (Figure 3A,B). In contrast, liver NK cells primarily clustered separately with only a few similarities between ascites and liver NK cells (Figure 3A,B). In line with previous literature [21, 32], liver NK cells expressed high levels of Eomes, CXCR6, CD69, and CCR5 while lacking CD16. Supporting these findings, also in conventional analysis, the subset of liver NK cells that are resident to the tissue (CXCR6^+^CD16^−^) was selective to this compartment and absent in the other tissues (Figure S3C,D). Interestingly, a cluster of NK cells could be assigned specifically to ascites. These cells were characterized mainly by expression of CD103, but also of other tissue‐residency markers such as CXCR6, CD69, and CD49a (Figure 3B). When analyzing CD56^dim^ and CD56^bright^ NK cells separately, this ascites‐specific cluster appeared more prominent in CD56^bright^ NK cells (Figure S2A,B). In an analysis of Phenograph clusters, ascites NK cells formed three separate clusters that all were marked by expression of CD103 (Figure 3C–E). Of interest, these CD103 expressing subsets displayed diverse phenotypes marked by differential expression of panKIR, CD16, and/or NKG2A (Figure 3E). Furthermore, two of the clusters expressed CD49e otherwise only found on peripheral blood NK cells (Figure 3E).

Collectively, high‐dimensional analysis shows separate clustering of blood, ascites, and liver NK cells and uncovers the presence of NK cells exhibiting tissue‐residency phenotypes in the ascites.

### 2.4 CD103^+^ NK Cells in Ascites Are a Heterogeneous Group of Tissue‐Resident NK Cells

**FIGURE 4:**
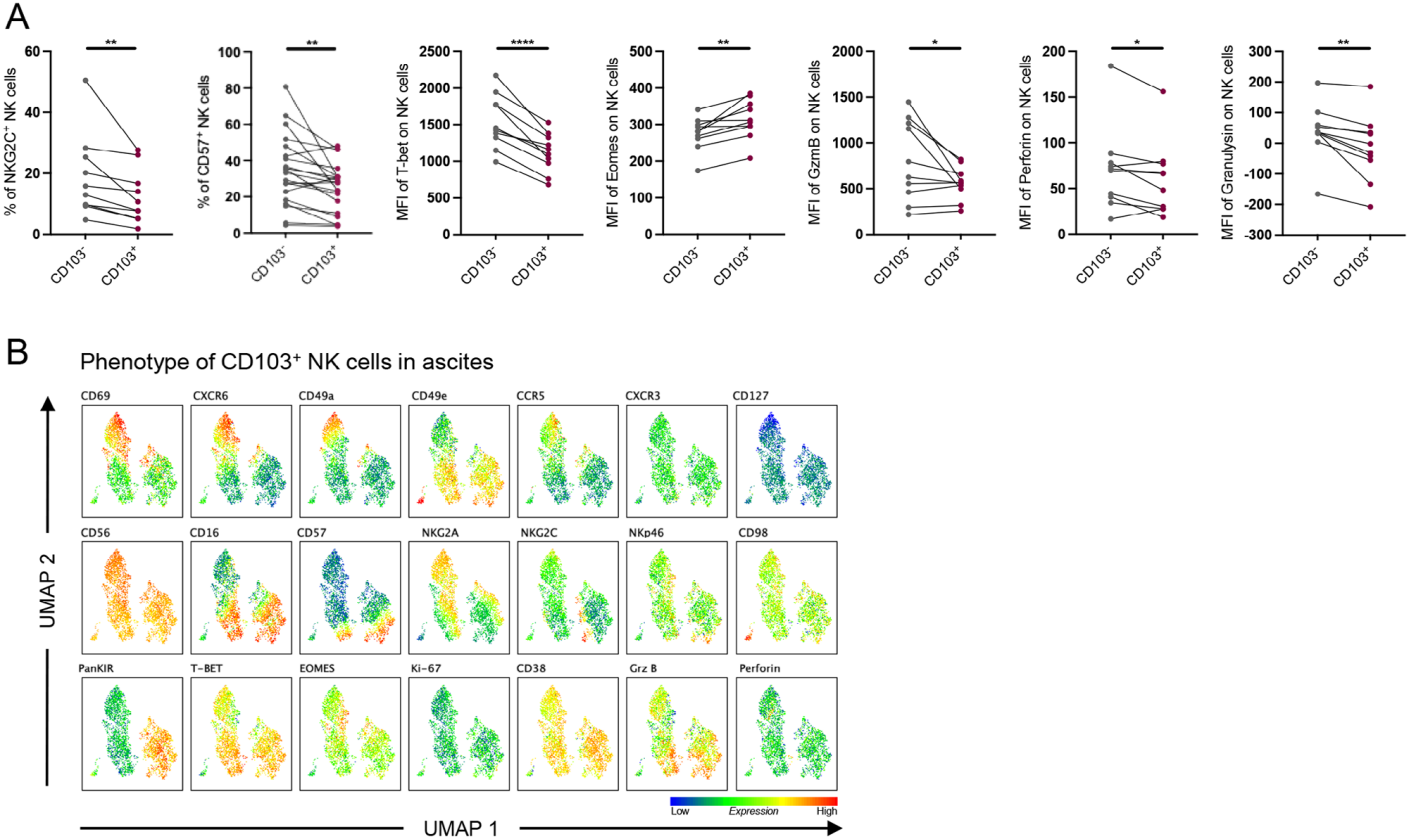
CD103^+^ NK cells in the ascites represent a heterogeneous group of NK cells. (A) Expression of indicated markers on CD103^+^ NK cells compared with their CD103^‐^ counterpart (*n* = 10–20) in ascites. (B) UMAP analysis depicting the phenotype of CD103^+^ ascites NK cells generated via concatenated (*n* = 10) CD103^+^ NK cells derived from the ascites. A paired *t*‐test was used to identify significance for normally distributed values, whereas the Wilcoxon test was performed on nonparametric datasets. *p < 0.05; **p < 0.01; ***p < 0.001; ****p < 0.0001.

Next, we set off to study CD103^+^ NK cells in ascites more in detail. First, we compared CD103^+^ NK cells to their CD103^−^ counterparts in ascites (Figure 4A; Figure S3A,B). In line with our previous observation that more CD103^+^ were enriched within the CD56^bright^ NK cell compartment, ascites‐derived CD103^+^ NK cells displayed a less differentiated, tissue‐resident phenotype compared with CD103^‐^ NK cells (Figure 4A; Figure S3A,B). Expression of NKG2C and CD57 was present but significantly reduced on CD103^+^ NK cells (Figure 4A). In addition, we noted lower expression of the transcription factor T‐bet and higher expression of Eomes, identifying these CD103^+^ cells as NK cells in contrast to innate lymphoid cells (ILCs) [33] (Figure 4A). Furthermore, ascites CD103^+^ NK cells had significantly lower levels of the effector molecules perforin, granzyme B, and granulysin compared with matched CD103^−^ NK cells (Figure 4A). Of note, the expression of the tissue‐residency/‐homing markers CXCR6, CCR5, and CD49a were higher on CD103^+^ NK cells compared with CD103^−^ NK cells (Figure S3A). Furthermore, Ki‐67 expression of CD103^+^ NK cells was comparable to that of CD103^‐^ counterparts (Figure S3B). In the UMAP analysis of ascites CD103^+^ NK cells, the cells clustered mainly according to CD49e, panKIR, and NKG2A expression (Figure 4B). Strikingly, despite all here analyzed cells being CD103^+^, the expression of cytotoxicity‐related markers CD16 and granzyme B and tissue‐residency markers such as CD69, CXCR6, and CD49 were to a large extent mutually exclusive, indicating different levels of tissue‐residency/functionality (Figure 4B).

In summary, ascites harbor a heterogeneous subset of CD103^+^ NK cells that predominantly exhibit a less differentiated, tissue‐resident phenotype.

### 2.5 CD103^+^ NK Cells Can be Generated Through Cytokines Present in the Ascites

**FIGURE 5:**
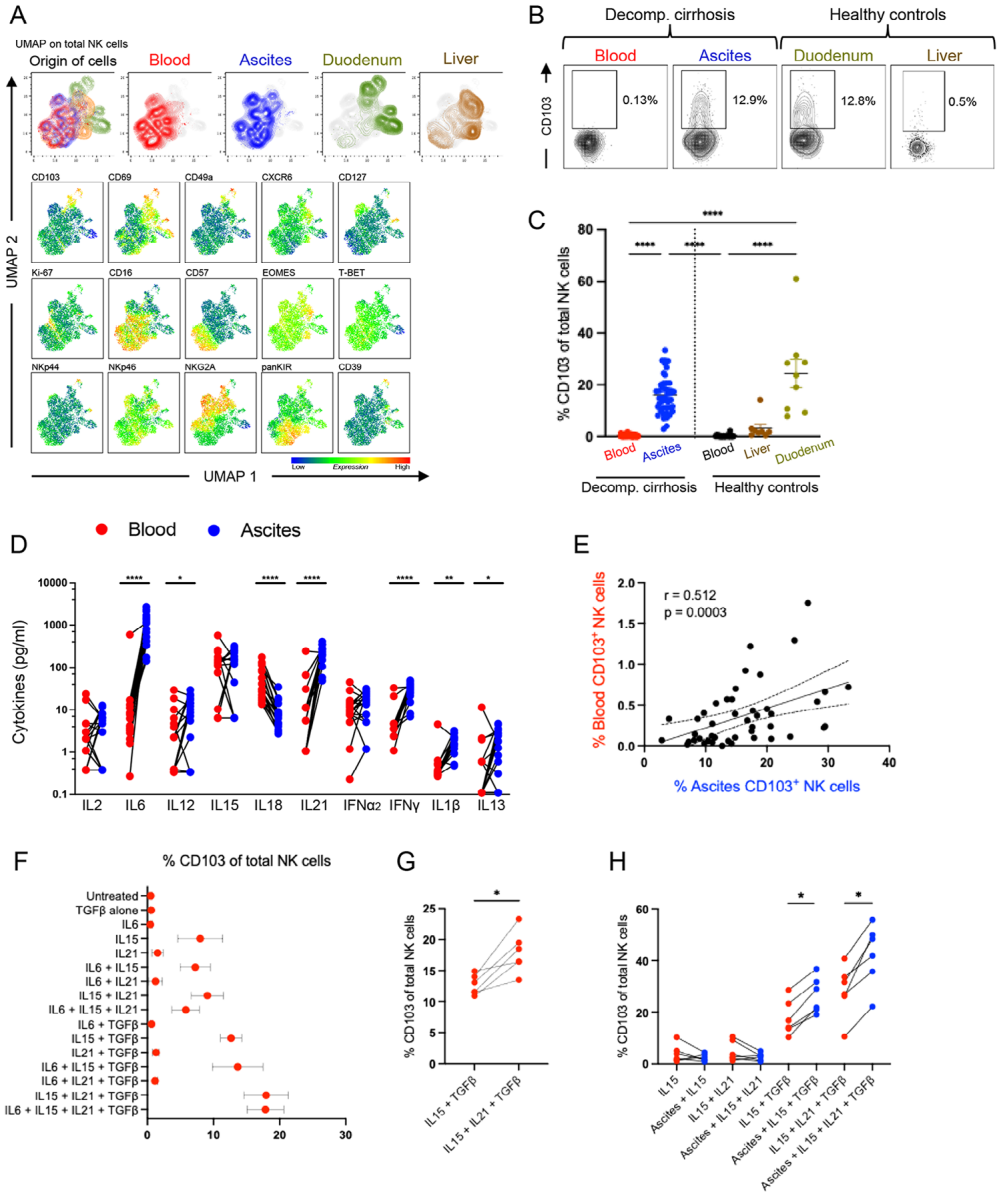
CD103^+^ NK cells can be generated through cytokines present in the ascites. (A) UMAP analysis of the total NK cell population stratified for sample origin (blood, ascites, duodenum, liver) and single marker plots identifying the main phenotypic clusters. Matched blood and ascites are derived from patients with decompensated liver cirrhosis and duodenum and liver samples from unmatched controls. UMAP analysis was performed after downsampling and concatenation of files. (B) Representative FACS plots showing CD103 expression on NK cells from different origins (blood, ascites, duodenum, liver). (C) Frequency of CD103^+^ NK cells in matched blood and ascites from patients with decompensated liver cirrhosis (*n* = 45) compared with blood (*n* = 23), liver (*n* = 8), and duodenum (*n* = 9) from healthy controls. (D) Abundance of the indicated cytokines (pg/mL) in matched blood and ascites from patients with decompensated liver cirrhosis (*n* = 16–18). (E) Correlation between the relative abundance of CD103^+^ NK cells in matched blood and ascites (*n* = 45). (F) Overview and (G) representative frequency of CD103^+^ among total NK cells after stimulation of peripheral blood NK cells from healthy controls (*n* = 6) for 5 days with the indicated cytokine combinations. (H) Frequency of CD103^+^ NK cells after stimulation of peripheral blood NK cells from healthy controls for 5 days in culture media supplemented with indicated cytokines and, when indicated, 50% pooled ascites supernatant. Paired *t*‐tests were used to identify significances for normally distributed values, whereas the Wilcoxon test was performed on nonparametric datasets. For multiple comparisons of matched nonparametric data, the Friedman test was performed. Spearman tests were used for the correlation between blood and ascites CD103^+^ NK cell frequencies. *p < 0.05; **p < 0.01; ***p < 0.001; ****p < 0.0001.

We next sought to identify mechanistic aspects of the heterogeneous phenotype of CD103^+^ NK cells in the peritoneal cavity as these cells were absent in the blood and liver. We hypothesized that the abundance of CD103^+^ NK cells in the ascites might be due to an increase in intestinal permeability and that CD103^+^ NK cells may originate from the intestine, as it has recently been shown that the intestine harbors NK cells identified by CD103 expression [19, 25]. To address this, we analyzed NK cells from the duodenum of healthy controls and compared the phenotype with NK cells from blood and ascites from patients with decompensated liver cirrhosis, as well as from liver tissue as control (Figure 5A–C; Figure S3E). Of note, duodenal and liver‐derived NK cells clustered largely separately while ascites NK cells displayed a large overlap with peripheral blood (Figure 5A). Of note, the frequency of CD103^+^ NK cells was comparable in ascites and duodenum NK cells while mostly absent in blood and liver NK cells (Figure 5B,C). Duodenal origin was marked by high expression of the tissue‐residency markers CD69 and CD49a while ascites NK cells expressed high levels of CD57 (Figure 5A; Figure S3E). Lastly, NKp44, a marker for intra‐epithelial innate lymphoid cells (ieILCs) [34] was only found on a few, predominantly duodenal NK cells (Figure 5A).

Given that certain phenotypes of CD103^+^ NK cells, such as co‐expression of panKIR and/or CD57, were exclusively found in ascites (Figure 3D; Figure S3E), we investigated if the local immune milieu present in the ascites might drive the expression of CD103. Thus, we studied several NK cells activating and proinflammatory cytokines indicating intestinal epithelial barrier dysfunction in plasma and matched ascites supernatant from patients with decompensated liver cirrhosis (Figure 5D). Indeed, the proinflammatory cytokines IL‐6, IL‐12, IL‐21, IFNγ, IL‐1β, and IL‐13, which have previously been shown to be associated with higher intestinal permeability and decreased intestinal barrier function [35, 36, 37], were markedly elevated in the peritoneal cavity compared with peripheral blood (Figure 5D). As TGFβ is a major driver for CD103 expression [38], we also assessed TGFβ levels in matched blood and ascites (Figure S3F). Of note, TGFβ levels were found to be significantly lower in ascites compared with matched peripheral blood (Figure S3F). Despite a low prevalence in circulation, the frequencies of CD103^+^ NK cells correlated positively in matched blood and ascites (Figure 5E). Of interest, frequencies of ascites NK cells and ascites CD103^+^ NK cells did not correlate with clinical parameters of disease severity indicated by bilirubin, AST, and ALT levels, respectively (Figure S3G).

Finally, we asked if the phenotype of CD103^+^ NK cells could be induced by different cytokines and/or the ascites milieu present in the peritoneal cavity of patients with decompensated liver cirrhosis. It has previously been reported that the expression of CD103 on NK cells is induced by the combination of IL‐15 and TGFβ[39, 40, 41], whereas IL‐21 has been shown to synergize with IL‐15 to enhance NK cell functionality [42]. Therefore, we studied the upregulation of tissue residency markers via stimulation with combinations of IL‐6, IL‐15, IL‐21, and TGFβ (Figure 5F). As expected, we observed an increased expression of CD103 on NK cells after joint stimulation with IL‐15 and TGFβ (Figure 5F). Of interest, IL‐21 acted synergistically with IL‐15 and further increased CD103 expression while IL‐6 had no additional effect (Figure 5F,G). Looking at other tissue‐residency markers, CD49a was upregulated most after joint stimulation with IL‐15, IL‐21, and TGFβ, whereas expression of CD69 was predominantly driven by IL‐15 (Figure S4A,B). Furthermore, ascites supernatant appeared to further facilitate CD103 expression in the presence of IL‐15, TGFβ, and IL‐21 (Figure 5H; Figure S4C). This was more pronounced on immature NK cell subsets but lower levels of CD103 could also be induced on differentiated NKG2A^‐^panKIR^+^CD57^+^ NK cells (Figure S4D).

In conclusion, CD103^+^ NK cells in ascites may be translocated either by passive influx from surrounding tissue or the bloodstream due to portal hypertension. Additionally, CD103 expression can be induced by the ascites cytokine milieu.

### 2.6 Peritoneal NK Cells Display Altered Functionality Compared with Blood and Liver NK Cells

**FIGURE 6:**
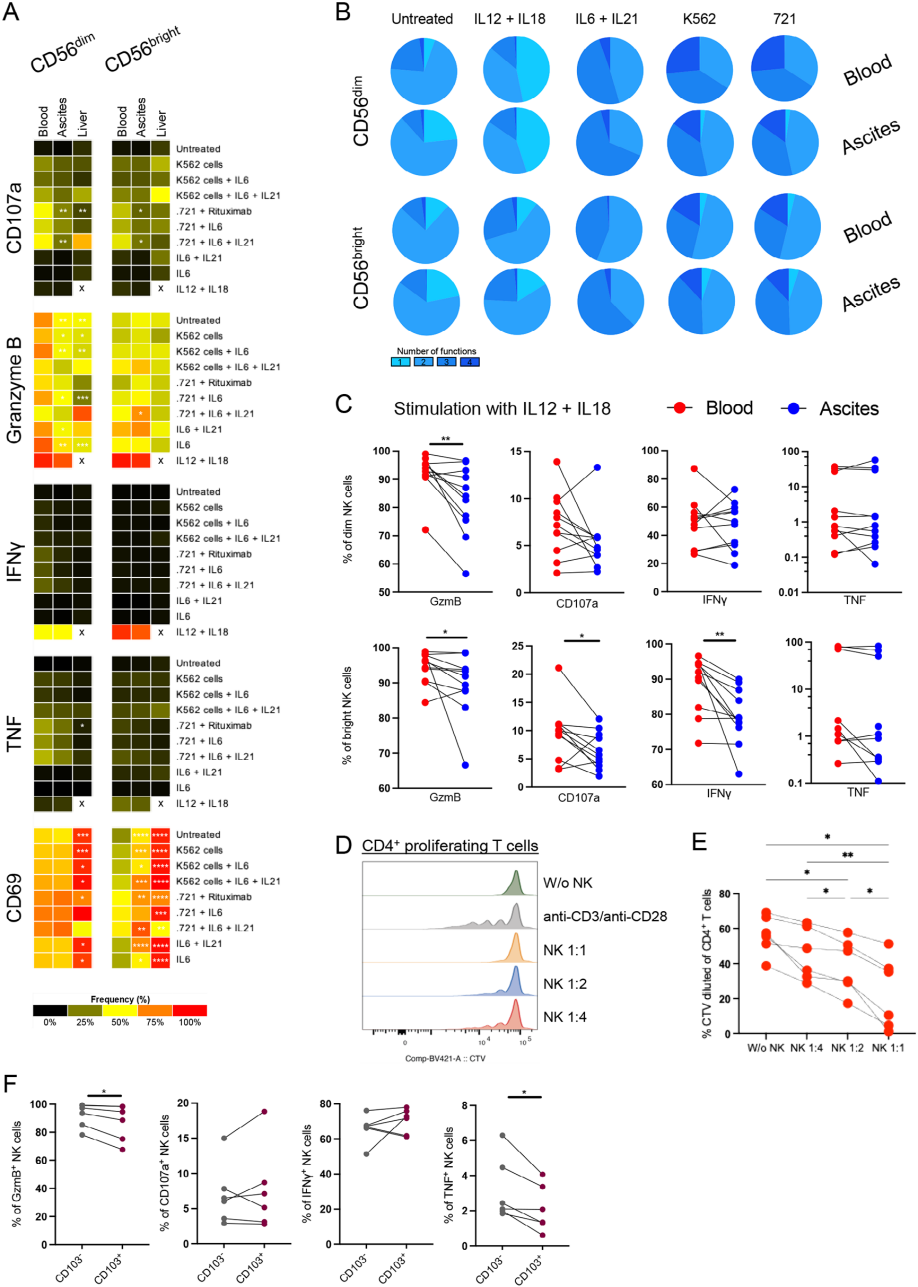
Peritoneal NK cells display altered functionality compared with blood and liver NK cells. (A) Heatmap summarizing the median frequency of responding cells for the indicated functional readouts following stimulation with different reagents for matched blood and ascites (*n* = 10) from patients with decompensated liver cirrhosis and liver (*n* = 5) samples from unmatched controls. (B) Multifunctional analysis of CD107a, Granzyme B, IFNγ, TNF, and CD69 responses for the indicated stimulation in matched blood and ascites NK cells; color denotes the number of simultaneously exhibited functions. (C) NK cell response following stimulation with IL‐12 + IL‐18 in matched blood and ascites samples (*n* = 11). (D, E) The proliferation of purified autologous peripheral blood T cells was assessed after anti‐CD3/anti‐CD28 stimulation for 5 days in the presence or absence of enriched primary ascites NK cells (*n* = 6). (F) Functional readout following stimulation with IL‐12 + IL‐18 of ascites CD103^+^ NK cells compared with ascites CD103^‐^ NK cells (*n* = 6). Functional readouts of paired samples were compared using paired *t*‐test or Wilcoxon test when appropriate. For multiple comparisons, the Kruskal–Wallis test was performed. *p < 0.05; **p < 0.01; ***p < 0.001; ****p < 0.0001.

As we observed the impact of the ascites cytokine milieu on NK cell phenotype, we next investigated the functional capacity of ascites NK cells in comparison to matched blood NK cells, and liver‐derived NK cells as reference. To this end, we studied global NK cell functionality by assessing cytotoxicity, antibody‐dependent cellular cytotoxicity (ADCC), and cytokine response. Given the increase of the proinflammatory cytokines IL‐6 and IL‐21 in the peritoneal cavity (Figure 5B) and their effect on NK cell functionality [42, 43], we included these cytokines for mimicking the cytokine milieu in ascites and to assess their effect on functionality. As expected, the profile of the NK cell response depended on the type of stimulation and the NK cell subset (CD56^bright^ vs. CD56^dim^) (Figure 6A). In general, we observed comparable NK cell cytotoxicity and multifunctionality in CD56^bright^ and CD56^dim^ subsets and tissues (Figure 6A,B). Yet, ascites and liver NK cells displayed an altered functional response profile compared with circulating NK cells, marked by reduced degranulation when assessing ADCC or after stimulation with IL‐6 and IL‐21 (Figure 6A). Furthermore, upon stimulation with IL‐12 + IL‐18, ascites NK cells were hyporesponsive and less functional compared with matched blood NK cells, indicated by lower Granzyme B, CD107a, and IFNγ production (Figure 6C). To investigate whether ascites NK cells interact with stimulated T cells in the peritoneal cavity, we studied the potential of ascites NK cells to act on autologous T‐cell proliferation. Indeed, ascites NK cells suppressed autologous CD4^+^ T cells in vitro and limited CD4^+^ T cell proliferation in a ratio‐dependent manner (Figure 6D,E). Lastly, given the distinct cluster of CD103^+^ NK cells in ascites, we assessed their functionality in more detail. Indeed, CD103^+^ NK cells derived from the ascites exhibited lower functional potential compared with CD103^‐^ NK cells (Figure 6F).

Collectively, these results suggest that NK cells, particularly CD103^+^ NK cells, from the ascites of patients with decompensated cirrhosis exhibit altered functionality in conjunction with inhibitory properties.

## 3 Discussion

Ascites is one hallmark of decompensated liver cirrhosis. In these patients, the intestinal and vascular barrier have become inefficient, and fluid accumulates in the peritoneal cavity, with a high risk of infection [7]. Here, we performed a comprehensive analysis of the innate immune milieu in the ascites by assessing NK cell phenotype, function, and cytokine profile in the ascitic fluid. We show that ascites NK cells are a heterogeneous group of cells with altered functionality and that CD103 expression identifies a subpopulation that is likely to be induced by cytokines present in the ascites fluid.

Liver cirrhosis leads to elevated pressure in the portal‐venous system which, in turn, contributes to the accumulation of ascites. In a previous study, we could show that mucosal‐associated invariant T cells are found at higher frequencies in ascites and that they are fully functional [44]. In line with this, we here report that also NK cells are found in the ascites at higher frequencies compared with peripheral blood, indicating a shift toward immune cells that are involved in early host protection. The comparison of ascites NK cell phenotype to blood and liver unveiled a subset of CD103^+^ NK cells, which constituted, on average, 15% of the total NK cell population in the ascites. This is in line with a previous report showing that CD103^+^ NK cells are present in the ascites of patients with chronic liver disease [27]. One cell type very similar to the here observed CD103^+^ NK cells is ieILCs [33, 34, 45]. Yet, we could show that only a few CD103^+^ cells in ascites express NKp44, a marker for ieILC [34, 45]. Consistent with this, ascites CD103^+^ NK cells expressed the transcription factor Eomes at higher, and T‐bet at lower levels which differs from the phenotype of intestinal ieILCs derived from the small intestine as recently described by Jaeger et al. [45]. In addition, previous reports have described bona fide CD103^+^ NK cells in the intestine [19, 25], supporting our findings.

Given the high frequency of NK cells in the ascites, it is intriguing to understand this enrichment. It can be speculated that the cells are either following the ascites with fluid due to the elevated portal‐venous pressure. The majority of NK cells in ascites displayed a phenotype comparable to peripheral blood, suggesting these cells originate from circulation. However, we observed that a subset of CD103^+^ NK cells in the ascites also showed similarities to cells found in the duodenum. This was further corroborated by high levels of several cytokines in ascites associated with intestinal epithelial barrier dysfunction [46], as well as the correlation of CD103^+^ NK cells from blood and ascites, suggesting an undirected passive translocation to these sites due to heightened intravascular pressure and leaky epithelia. On the other hand, CD103^+^ NK cells in the ascites could also simultaneously express CD103, KIR, and/or CD16, a combination not found in other tissues. Furthermore, we could show that IL‐21 can further increase IL‐15 and TGFβ‐mediated CD103 upregulation and that this is further facilitated by ascites supernatant. Of note, levels of TGFβ were found to be reduced in cirrhosis‐associated ascites. This is in line with a previous report investigating NK cells in malignant ascites [47]. However, given the phenotype observed in ascites NK cells, we argue that local concentrations of TGFβ in ascites might be sufficient to induce CD103 expression. Collectively, these data suggest that NK cells may passively translocate predominantly from the bloodstream to the ascites and that the local cytokine milieu in the ascites fluid induces CD103 upregulation.

Given the advanced cirrhosis and high portal‐venous pressure, one could also speculate that liver‐resident NK cells are found in the ascites of patients with decompensated liver cirrhosis. However, we show here that only a few, if any, NK cells with a liver‐specific phenotype are found in the ascites.

Furthermore, recent studies on malignant ascites from ovarian cancer patients highlight the importance of NK cells in the ascites [48, 49, 50, 51]. Bernson et al. [52] recently reported that CD103^+^ NK cells exist in malignant ascites from ovarian cancer patients at similar levels as observed here, and Chung et al. [41] showed that TGFβ in ascites supplemented with IL‐15 is a major driver for the expression of CD103 in malignant ascites. However, in line with a recent study showing that malignant ascites contain increased levels of TGFβ compared with nonmalignant ascites [47], it is likely that the malignant and nonmalignant ascites have quite diverse impacts on NK cells despite similar NK cell phenotypes. The environment in the ascites also appears to affect NK cell functionality, as we found reduced ADCC and cytokine response compared with peripheral blood. Furthermore, ascites NK cells were able to inhibit autologous T cell proliferation. These observations on NK cell functionality are in line with the findings by Fraser et al. [48] and Chung et al. [41] that showed suppressed NK cell function and inhibition of T cell proliferation mediated by induced CD103^+^ NK cells. Furthermore, Fraser et al. [48] describe an alteration of the transcriptional profile of ascites NK cells indicating a downregulation of NK cell function [48]. Hence, it can be speculated that the ascites environment both in malignant and nonmalignant affects NK cell functionality.

Of note, a limitation of our study is the lack of matched liver and duodenum samples from patients with decompensated liver cirrhosis and ascites. Additionally, duodenal samples do not reflect all intestines, and cells contained in the jejunum, ileum, or colon might be different. Moreover, future studies should address what other factors in ascites mediate the expression of CD103.

In conclusion, we show that expression of CD103 identifies a subset of NK cells in the peritoneal cavity that is distinct from NK cells in the liver and blood and is likely to be induced by the local cytokine milieu. Furthermore, the NK cells that reside in the peritoneal cavity display an altered functionality in conjunction with the capacity for inhibitory properties. Consequently, these cells may take on a regulatory role within the peritoneal cavity, mirroring alterations in the ascites immune compartment.

## 4 Materials and Methods

### 4.1 Study Design and Sample Collection

A total of 70 patients with liver cirrhosis were enrolled in this study. The underlying causes of liver cirrhosis were either alcohol‐related or chronic infection with hepatitis viruses. Fifty‐nine patients with decompensated liver cirrhosis were recruited in a prospective trial (DRKS00010664) (Figure 1A). From these patients, peripheral blood mononuclear cells (PBMC) and plasma were acquired from peripheral blood, and mononuclear cells (MNCs) and supernatant were collected from ascites. Further, 11 patients with compensated cirrhosis were recruited and sampled for PBMC and plasma. Hepatocellular carcinoma, human immunodeficiency virus infection as well as ongoing spontaneous bacterial peritonitis (SBP) were exclusion criteria. All patients were seen and recruited at the Department of Gastroenterology, Hepatology, and Endocrinology at Hannover Medical School. From all participants, oral and written informed consent was obtained. The Ethics Committee of Hannover Medical School approved this study (3188‐2016) and the study protocol is in accordance with the ethical guidelines of the 1975 Declaration of Helsinki. As controls, peripheral blood samples from healthy volunteers (*n* = 33) were collected as well as samples from liver tissue (liver MNCs, *n* = 8) as previously described [21, 53]. Liver samples were acquired from the unaffected part of the liver during liver resection (cause for resection colorectal metastasis, *n* = 5; adenoma, *n* = 1; unknown/suspected carcinoma, *n* = 2). Duodenal samples (*n* = 9) were collected from patients during pancreatoduodenectomy for intraductal papillary mucinous neoplasm (premalignant condition). Samples were then taken from the surgically removed duodenum that was considered medical waste. Both liver and duodenal samples were collected at Karolinska University Hospital, Stockholm, Sweden (2013/2285‐31/3), after written informed consent was obtained. The study outline and workflow are depicted in Figure 1A. Patient characteristics are detailed in Table S1.

Ascites samples were obtained during paracentesis and matched peripheral blood was collected during routine blood sampling. Subsequently, PBMCs were obtained from fresh whole blood via Ficoll‐density gradient centrifugation and ascites MNCs were isolated by centrifugation (5 min at 800*g*) followed by lysis of red blood cells. Then, cells were cryopreserved in liquid nitrogen for later analysis. Plasma and ascites supernatant were obtained from EDTA blood or ascites samples after centrifugation and collection of supernatants before being stored at −80°C until further use. Liver tissue samples were processed, and mononuclear cells were isolated as previously described [21]. Duodenal samples were cut into small pieces before being digested for 30 min at 37°C in RPMI 1640 containing 10% FCS, 1 mM l‐glutamine, and collagenase II at 0.25 mg/mL and DNase at 0.2 mg/mL. The obtained cell suspension was then filtered through a 70 mm cell strainer and mononuclear cells were isolated via Ficoll‐density centrifugation.

### 4.2 Flow Cytometry

Cryopreserved MNCs and PBMCs were thawed and stained with fluorochrome‐labeled monoclonal antibodies as previously described [44, 54]. In brief, cryopreserved PBMCs and MNCs were thawed in complete RPMI medium (RPMI supplemented with 10% FCS and 2 mM L‐glutamine) and washed twice in FACS buffer (PBS + 2%FCS + 2 mM EDTA) before surface and intracellular staining with the desired antibody mixture was performed at room temperature in the dark for 15 and 30 min, respectively. For the exclusion of dead cells, the samples were stained with either Live Dead Fixable Aqua, Live Dead Fixable Green, or Fixable Viability Stain 700. Fixation/permeabilization was performed with eBioscience Fixative (FOXP3/Transcription Buffer staining set, eBioscience). Antibodies used for staining are listed in Table S2. Samples were measured on a 14‐ or 18‐color LSR Fortessa flow cytometer as well as on a 30‐color BD symphony flow cytometer (all BD Biosciences). Data were analyzed with FlowJo v10.5.3. High‐dimensional analysis was performed via UMAP [55] analysis and clustering using Phenograph [56] with publicly available FlowJo plugins as previously described [44].

### 4.3 Functional NK Cell Assays and Analysis

Different stimuli were used to assess NK cell functionality. NK cell cytotoxicity was investigated via the addition of K562 cells at an effector‐to‐target ratio of 10:1 for 6 h; cytokine responses were assessed after adding IL‐12 (10 ng/mL) and IL‐18 (100 ng/mL) for 18 h; ADCC was measured by addition of 721.221 cells at a 10:1 effector to target ratio together with 1 µg/mL Rituximab for the last 6 h. IL‐6 (10 ng/mL) and IL‐21 (10 ng/mL) were either used for the stimulation alone or combined with K562 cells or 721.221 cells, respectively. CD107a, Brefeldin A, and Monensin were added for the last 5 h of stimulation. In the indicated experiments, NK cells were enriched from peripheral blood with the NK cell isolation kit (Miltenyi) according to the manufacturer's instructions and 1–2*10^5^ cells/well were stimulated for five days with different combinations of cytokines (10 ng/mL IL‐6, 10 ng/mL IL‐15, 10 ng/mL IL‐21, 10 ng/mL TGFβ) with 200 µL media/well with or without pooled ascites supernatant (100 µL media + 100 µL ascites supernatant per well). Additionally, inhibition with monoclonal antibodies targeting IL‐21 (Biotechne, 1 ug/mL) was utilized in blockade experiments. Culture media was renewed after two days. As a control, from each donor, bulk PBMCs were treated with the same conditions. Subsequently, cells were stained for flow cytometry as described above. Multifunctional responses were analyzed with SPICE version 6 (National Institutes of Health, Bethesda, MD) [57]. To this end, boolean gates of NK cells expressing Granzyme B, CD107a, TNF, and/or IFN‐γ were used and respective frequencies were extracted.

### 4.4 T Cell Proliferation Assay

To assess the effect of ascites NK cells on T cell proliferation, T cells were enriched from peripheral blood PBMCs with the pan T cell isolation kit (Miltenyi) following the manufacturer's instructions. 1*10^5^ enriched T cells/well were activated with anti‐CD3/anti‐CD28 (10 and 2.5 µg/mL, respectively) and labeled with CellTrace Violet (Thermofisher). Labeled T cells were then co‐cultured with NK cells isolated from the ascites fluid using the NK cell isolation kit (Miltenyi). The experiment was performed in a 1:1, 1:2, and 1:4 T cell to NK cell ratio and incubated for five days. As controls, T cells were left unstimulated or stimulated without NK cells. The cells were then stained and analyzed as described above.

### 4.5 Cytokine Measurement

Plasma and ascites supernatant samples were measured in one run with the LUMINEX‐based multiplex bead assay (Human Cytokine Assay; 171AA001M; Bio‐Rad, USA) according to the manufacturer's instructions together with optimized protocols. For acquisition, the BioPlex Manager 6.0 software was used. Furthermore, a TGFβ1‐ELISA (Human TGF beta‐1 ELISA Kit; BMS249‐4; Thermofisher) was performed according to the manufacturer's instructions on matched ascites and blood samples from patients with decompensated liver cirrhosis and peripheral blood samples from healthy controls.

### 4.6 Statistical Analysis

For statistical analyses, GraphPad Prism software 9.0 was used. In the first step, the distribution of datasets was tested with the D’ Agostino & Pearson normality test. For the comparison of two unmatched but normally distributed groups, an unpaired *t*‐test was applied, while the Mann–Whitney test was used for not normally distributed datasets. In the case of matched, normally distributed samples a paired *t*‐test was performed while a Wilcoxon matched‐pairs signed rank test was used for not normally distributed samples. For comparison of more than two datasets, either Kruskal–Wallis or one‐way analysis of variance (ANOVA) was performed when indicated. Pearson's or Spearman's *r* coefficients were calculated for analysis of the correlation between datasets. Statistical tests are detailed in the respective figure legends. Significances in the graphs are displayed as *p < 0.05; **p < 0.01; ***p < 0.001; ****p < 0.0001.

## Authors Contributions

All authors contributed to the study as follows. Christian Niehaus, Benedikt Strunz, and Markus Cornberg planned the study. Christian Niehaus, Benedikt Strunz, Christine S. Falk, Daniel Geanon, Christopher Maucourant, Ayesha Lietzau, and Marija Jankovic performed the experiments. Christian Niehaus, Benedikt Strunz, Daniel Geanon, Anke R.M. Kraft, Niklas K. Björkström, Markus Cornberg, Ayesha Lietzau, and Marija Jankovic analyzed the data. Christian Niehaus, Benedikt Strunz, and Markus Cornberg wrote the manuscript. Christian Niehaus, Markus Cornberg, Benjamin Maasoumy, Heiner Wedemeyer, Niklas K. Björkström, Itzel Medina, Ernesto Sparrelid, Julia Kahlhöfer, and Andrea Ponzetta contributed to patient recruitment and/or sample collection. All authors contributed to critical revisions and approved the final manuscript.

## Conflicts of Interest

BM served as a speaker and/or advisory board member for AbbVie, AstraZeneca, EWIMED, Fujirebio, Gilead, Luvos, MSD, Norgine, Roche, and W. L. Gore & Associates and received research support from Altona, EWIMED, Fujirebio, and Roche. MC reports receiving lecture and/or consulting fees from AbbVie, AiCuris, AstraZeneca, Falk, Gilead, GSK, MSD Sharp & Dohme, and Roche, outside the scope and topic of this study. HW served as a speaker / advisory board member for Abbott Laboratories & Abbott Molecular, Albireo Pharma, AstraZeneca, Atea Pharmaceuticals, Bristol‐Myers‐Squibb, Dr. Falk Pharma, Hoffmann‐La Roche, Gilead Sciences GmbH & Gilead Sciences Ltd., GlaxoSmithKline Services Unlimited, Janssen, Lilly Deutschland, Mirum Pharmaceuticals, MSD Sharp & Dohme, Orphalan, Pfizer, Roche Diagnostics International, Sobi, Takeda, Vir Biotechnology, received research support from Abbott Laboratories & Abbott Molecular, Biotest AG and received lecture fees from Biotest AG, BioMarin Pharmaceuticals, CSL Behring, Falk Foundation and Olink. The remaining authors declare no conflicts of interest.

## Peer Review

The peer review history for this article is available at https://publons.com/publon/10.1002/eji.202451311.

## Supporting information



Supporting information

## Data Availability

The data that support the findings of this study are available from the corresponding author upon reasonable request.
