# Supplementary material for: Origin of Hypofunctional CD103+ NK Cells in Cirrhosis‐Associated Ascites
Source: Eur J Immunol. 2025 Jun 11;55(6):e51311. doi: 10.1002/eji.202451311 (PMC12154167; doi:10.1002/eji.202451311)

Supplementary material

Origin of hypofunctional CD103$^+$ NK cells in cirrhosis-associated ascites

Christian Niehaus, Daniel Geanon, Ayesha Lietzau, Marija Jankovic, Christopher Macourant, Benjamin Maasoumy, Ernesto Sparrelid, Heiner Wedemeyer, Julia Kahlhöfer, Christine S. Falk, Itzel Medina Andrade, Andrea Ponzetta, Niklas K. Björkström, Anke RM Kraft, Markus Cornberg*, Benedikt Strunz*

Table of contents

Supplementary Table S1

Supplementary Table S2

Supplementary figure legends

Supplementary Figure 1

Supplementary Figure 2

Supplementary Figure 3

Supplementary Figure 4

Supplementary Table S1. Patient characteristics of all patients included in this study.

Parameter	Healthy	Compensated cirrhosis	Decompensated cirrhosis
**Number of patients**	50	11	59
**Gender**			
- Female	n/a	6	23
- Male	n/a	5	36
**Age**	56.5 ±9.9	58.1 ±14.8	64.3 ±8.9
**AST (U/L)**	/	85.4 ±58.2	53.5 ±39
**ALT (U/L)**	/	105.5 ± 127.7	26.4 ±21.6
**Bilirubin (umol/L)**	/	12.4 ±6	52.6 ±86.3
**Albumin (g/dL)**	/	37.9 ±3.9	28.7 ±6.6
**INR**	/	1.2 ±0.2	1.4 ±0.4
**CRP (mg/L)**	/	4.4 ±3.6	29.9 ±36.1
**Etiology**			
- HCV	/	4	5
- HBV/HDV	/	7	5
- ARC	/	/	49

All values provided as mean ± SD
ALT, alanine transaminase; ARC, alcohol-related liver cirrhosis; AST, aspartate transaminase; CRP, C-reactive protein; INR, International Normalized Ratio.

Supplementary Table S2. Antibodies for flow cytometry.

Antibody name	Color	Clone	Supplier
anti-CCR2	FITC	K036C2	BioLegend
anti-CXCR1	PE-Cy7	8F1	BioLegend
anti-Ki-67	Alexa Fluor 488	Ki-67	BioLegend
anti-Ki-67	Brilliant Violet 711	Ki-67	BioLegend
anti-CD107a (LAMP-1)	Alexa Fluor 647	H4A3	BioLegend
anti-CD49a (Integrin α1)	Alexa Fluor 647	TS2/7	BioLegend
anti-CCR5 (CD195)	Alexa Fluor 647	HEK/1/85a	BioLegend
anti-Granulysin	Alexa Fluor 647	DH2	BioLegend
anti-CD45 (LCA)	Brilliant Violet 570	HI30	BioLegend
anti-CD45 (LCA)	Alexa Fluor 700	HI30	BioLegend
anti-CD103 (Integrin αE)	APC	Ber-ACT8	BioLegend
anti-CD57	APC	QA17A04	BioLegend
anti-CD57	Pacific Blue	HCD57	BioLegend

anti-CD8	APC-Fire750	RPA-T8	BioLegend
anti-CD4	Brilliant Violet 570	RPA-T4	BioLegend
anti-CD14	V500	M5E2	BioLegend
anti-CD279 (PD-1)	Brilliant Violet 421	EH12.2H7	BioLegend
anti-IFN-γ	Brilliant Violet 421	4S.B3	BioLegend
anti-IFN-γ	Brilliant Violet 785	4S.B3	BioLegend
anti-T-bet	PE-CF594	O4-46	BD Biosciences
anti-T-bet	Brilliant Violet 421	4B10	BioLegend
anti-CXCR6 (CD186)	Brilliant Violet 421	K041E5	BioLegend
anti-CD73	Brilliant Violet 421	AD2	BioLegend
anti-CD123	Brilliant Violet 510	6H6	BioLegend
anti CD3	Brilliant Violet 510	UCHT1	BioLegend
anti CD3	Brilliant Violet 750	17A2	BioLegend
anti-CD8	Brilliant Violet 605	SK1	BioLegend
anti-CD69	Brilliant Violet 786	FN50	BioLegend
anti-CD161	Brilliant Violet 605	HP-3G10	BioLegend
anti-CD161	APC-Cy7	HP-3G10	BioLegend
anti-CD16	Brilliant Violet 650	3G8	BioLegend
anti-HLA-DR	Brilliant Violet 650	L243	BioLegend
anti-TNF-α	Brilliant Violet 650	Mab11	BioLegend
anti-CD38	Brilliant Violet 650	HB-7	BioLegend
anti-CD14	Brilliant Violet 711	M5E2	BioLegend
anti-CD19	Brilliant Violet 711	HIB19	BioLegend
anti-CD19	APC/Fire 810	HIB19	BioLegend
anti-DNAM-1	APC/Fire 750	11A8	BioLegend
anti-CD3	Brilliant Violet 750	SK7	BioLegend
anti-Granzyme B	PE	QA16A02	BioLegend
anti-CD3	PE-Cy5	UCHT1	BioLegend
anti-CD19	PE-Cy5	HIB19	BioLegend
anti-CXCR4 (CD184)	PE-Cy5	12G5	BioLegend
anti-CD94	PE-Cy7	DX22	BioLegend
anti-CD127	PE-Cy7	A019D5	BioLegend
anti-CD127	Brilliant Violet 711	A019D5	BioLegend
anti-CD56	PE-Cy7	QA17A16	BioLegend
anti-CD69	PE-Dazzle594	FN50	BioLegend
anti-NKp46	Brilliant Violet 786	9E2/NKp46	BD Biosciences
anti-CD161	Brilliant Violet 650	DX12	BD Biosciences
anti-CD4	Brilliant Violet 750	SK3	BD Biosciences

anti-CD103 (Integrin αE)	FITC	Ber-ACT8	BD Biosciences
anti-CD103 (Integrin αE)	Brilliant Violet 711	Ber-ACT8	BD Biosciences
anti-CD103 (Integrin αE)	Brilliant Violet 785	Ber-ACT8	BD Biosciences
anti-CD103 (Integrin αE)	BB660	Ber-ACT8	BD Biosciences
anti-CD39	Brilliant Violet 605	TU66	BD Biosciences
anti-CD39	Brilliant Violet 711	TU66	BD Biosciences
anti-Perforin	BB755	δG9	BD Biosciences
anti-CD107a	FITC	H4A3	BD Biosciences
anti-CD16	APC-Cy7	3G8	BD Biosciences
anti-CD163	Alexa Fluor 647	GHI/61	BD Biosciences
Fixable Viability Stain 700			BD Biosciences
anti-CXCR3 (CD183)	Brilliant Violet 421	1C6	BD Biosciences
anti-CD3	Brilliant Violet 786	SK7	BD Biosciences
anti-CD3	Brilliant Ultraviolet661	UCHT1	BD Biosciences
anti-CD38	Brilliant Ultraviolet661	HIT2	BD Biosciences
anti-CD14	Violet 500	M5E2	BD Biosciences
anti-CD19	Brilliant Violet 510	SJ24C1	BD Biosciences
anti-CD15	Brilliant Violet 510	HI98	BD Biosciences
anti-CD98	Brilliant Violet 650	UM7F8	BD Biosciences
anti-CD56	PE-CF594	NCAM 16.2	BD Biosciences
anti-CD56	PE-Cy7	NCAM 16.2	BD Biosciences
anti-CD56	Brilliant Ultraviolet563	NCAM 16.2	BD Biosciences
anti-TNF-α	PE-CF594	Mab11	BD Biosciences
anti-CD49a (Integrin α1)	Brilliant Ultraviolet615	SR84	BD Biosciences
anti-CD49a (Integrin α1)	BB700	SR84	BD Biosciences
anti-CXCR6	BB630	13B1E5	BD Biosciences
anti-NKG2D	Brilliant Ultraviolet615	1D11	BD Biosciences
anti-NKG2D	Brilliant Ultraviolet737	1D11	BD Biosciences
anti-HLA-DR	Brilliant Ultraviolet395	G46-6	BD Biosciences
anti-CD8	Brilliant Ultraviolet395	RPA-T8	BD Biosciences
anti-CCR5	Brilliant Ultraviolet395	2D7	BD Biosciences

anti-CD16	Brilliant Ultraviolet737	3G8	BD Biosciences
anti-CD16	Brilliant Ultraviolet496	3G8	BD Biosciences
anti-CD16	Brilliant Violet 605	3G8	BD Biosciences
anti-CD69	Brilliant Ultraviolet395	FN50	BD Biosciences
anti-CD69	Brilliant Ultraviolet737	FN50	BD Biosciences
anti-Ki-67	Brilliant Ultraviolet395	B56	BD Biosciences
anti-Granzyme B	Alexa Fluor 647	GB11	BD Biosciences
anti-Granzyme B	BB790	GB11	BD Biosciences
anti-CD279 (PD-1)	Brilliant Ultraviolet737	EH12.1	BD Biosciences
anti-CD8	Brilliant Ultraviolet805	SK1	BD Biosciences
anti-CXCR3	Brilliant Ultraviolet805	1C6	BD Biosciences
anti-CCR6	APC-R700	11A9	BD Biosciences
Streptavidin	BB630		BD Biosciences
anti-Eomes	PE-eFluor 610	WD1928	ThermoFisher
anti-Eomes	VioBright FITC	WD1928	ThermoFisher
anti-CD19	PE-Cy5	HIB19	ThermoFisher
anti-CD8	QD605	3B5	ThermoFisher
Streptavidin	QD585		ThermoFisher
Live Dead Fixable Aqua			ThermoFisher
Live Dead Fixable Green			ThermoFisher
Live Dead Fixable Blue			ThermoFisher
anti-CD161	Biotin	191B8	Miltenyi
anti-CD14	APC	REA599	Miltenyi
anti-CD49e	VioBright FITC	REA686	Miltenyi
anti-CD107a	VioBright FITC	H4A3	Miltenyi
anti-NKG2C	Biotin	REA205	Miltenyi
anti-NKG2A	PE	REA110	Miltenyi
anti-CD57	APC-Vio770	TB03/REA769	Miltenyi
anti-CD57	PE	REA769	Miltenyi
anti-CD117 (c-kit)	PE-Cy5.5	95C3	Beckman Coulter
anti-CD56	PE-Cy5.5	N901	Beckman Coulter
anti-KIR2DL1/S1	PE-Cy5.5	EB6	Beckman Coulter
anti-KIR2DL2/L3/S2	PE-Cy5.5	GL183	Beckman Coulter

anti-NKp44	PE-Cy5.5	Z231	Beckman Coulter
anti-CD69	ECD	TP1.55.3	Beckman Coulter
anti-NKG2A (CD159a)	APC	Z199	Beckman Coulter
anti-CD103	Brilliant Ultraviolet395	Ber-ACT8	BD Biosciences
anti-CD8	Brilliant Ultraviolet563	RPA-T8	BD Biosciences
anti-CD56	Brilliant Ultraviolet737	NCAM16.2	BD Biosciences
anti-CD45	Brilliant Ultraviolet 805	HI30	BD Biosciences
anti-CXCR6	BB660	13B1E1	BD Biosciences
anti-CD69	APC	FN50	BioLegend
Cell trace violet			Invitrogen
anti-HLA-DR	Brilliant Violet 605	G46-6	BD Biosciences
anti-CD25	Brilliant Violet 711	BC96	BioLegend
anti-CD127	PE/Dazzle 594	A019D5	BioLegend
anti-Ki-67	Alexa Fluor 700	B56	BD Biosciences
anti-CD14	Brilliant Violet 510	M5E2	BioLegend
anti-CD8	Brilliant Violet 570	RPA-T8	BioLegend
anti-CD335 (NKp46)	Brilliant Violet 650	9E2	BioLegend
anti-CD16	Brilliant Violet 785	3G8	BD Biosciences
anti-NKp44	PE-Cy5	Z331	Beckman Coulter
anti-CD161	APC/Fire 750	HP-3G10	BioLegend
anti-CD94	BB700	HP-3D9	BioLegend
anti-CD57	Brilliant Violet 605	Qa1704	BioLegend
anti-CD39	Brilliant Violet 650	TU66	BD Biosciences
anti-CD69	Brilliant Violet 785	FM50	BioLegend
anti-NKG2A	PE-Cy7	Z199	Beckman Coulter

Supplementary Fig. 1. Gating strategy and conventional flow cytometry analysis of total NK cells.

(A) Gating of NK cells was performed as follows: First, time gating was performed to exclude clumps. This was followed by exclusion of doublet events and lymphocyte gating. This was followed by removal of dead cells and $CD14^{+}CD19^{+}$ cells and gating on CD3 negative cells. $CD56^{+}$ cells were then identified as NK cells and subdivided

into CD56^{bright} and CD56^{dim} NK cells. (B, C) Mean fluorescence intensity (MFI) and (C) frequencies of indicated phenotypic markers on total NK cells from matched blood and ascites from patients with decompensated liver cirrhosis (n=9-47), as well as liver samples from controls as a reference (n=5-8). For comparison of paired blood and ascites samples, paired t-test was used to identify significances for normally distributed values, whereas Wilcoxon test was performed on non-parametric datasets. * $p<0.05$; ** $p<0.01$; *** $p<0.001$; **** $p<0.0001$.

Supplementary Fig. 2. UMAP analysis of CD56^{dim} and CD56^{bright} NK cells.

(A, B) UMAP analysis of CD56^{dim} and CD56^{bright} NK cells from matched peripheral blood and ascites samples in patients from decompensated liver cirrhosis as well as liver samples from unrelated controls. After gating on CD56^{dim} and CD56^{bright} NK cells, respectively, equal number of cells were exported for peripheral blood, ascites, and liver samples (n=5 pairs for blood and ascites; n=5 liver samples). Expression of indicated markers are depicted as single marker plots and cells were distributed according to the origin.

Supplementary Fig. 3. Phenotype analysis of CD103^{+} NK cells and correlation with clinical markers of disease severity.

(A, B) Frequency or mean fluorescence intensity (MFI) of indicated tissue-resident (A) and phenotypic markers expressed on CD103^{+} NK cells from ascites compared with CD103^{-} ascites NK cells (n=10-20). (C) Frequency of liver-resident (CXCR6^{+}CD16^{-}) NK cells out of total NK cells in peripheral blood from patients with decompensated liver cirrhosis compared with matched ascites NK cells, and liver tissue from unmatched controls as a reference. Liver samples were run in a separate experiment. (D) Combinations of CXCR6$^{+/-}$ and/or CD16$^{+/-}$ out of CD103^{+} NK cells in matched

peripheral blood and ascites, as well as liver samples. (E) Comparison of indicated marker expression on unrelated duodenal and ascites CD103$^+$ NK cells (n=9 and 15, respectively). (F) Levels of TGFβ measured in matched blood and ascites from patients with decompensated liver cirrhosis (n=34) and in blood from healthy controls (n=10). (G) Correlation of frequencies of total ascites NK cells and CD103$^+$ NK cells with clinical markers of disease severity (Bilirubin, AST, and ALT) (n=39-45). For comparisons of matched samples, paired t-test was used to identify significances for normally distributed values, whereas Wilcoxon test were performed on non-parametric datasets. For multiple comparison, Kruskal Wallis test was performed. Pearson and Spearman-test were used when appropriate to correlate between variables. * $p<0.05$; ** $p<0.01$; *** $p<0.001$; **** $p<0.0001$.

Supplementary Fig. 4. Expression of tissue-residency markers on blood NK cells after co-incubation with different agents.

(A and B) Expression of CD49a (A) and CD69 (B) on enriched peripheral blood NK cells from healthy controls (n=6) after incubation for five days with the indicated combinations of cytokines. (C and D) Representative flow cytometry plots (C) and summary stratified for NK cell differentiation status (D) displaying the expression of CD103 on enriched NK cells from peripheral blood of healthy controls after incubation in media supplemented with indicated cytokines and, when indicated, 50% pooled ascites supernatant (n=6). (E) CD103 expression after incubation of enriched peripheral blood NK cells for 5 days with 50% pooled ascites supernatant in presence or absence of indicated cytokines and blocking antibody (n=6). For paired sample comparisons, Wilcoxon test was performed on non-parametric datasets. * $p<0.05$.

Supplementary Figure 1: Gating strategy and conventional flow cytometry analysis of total NK cells.

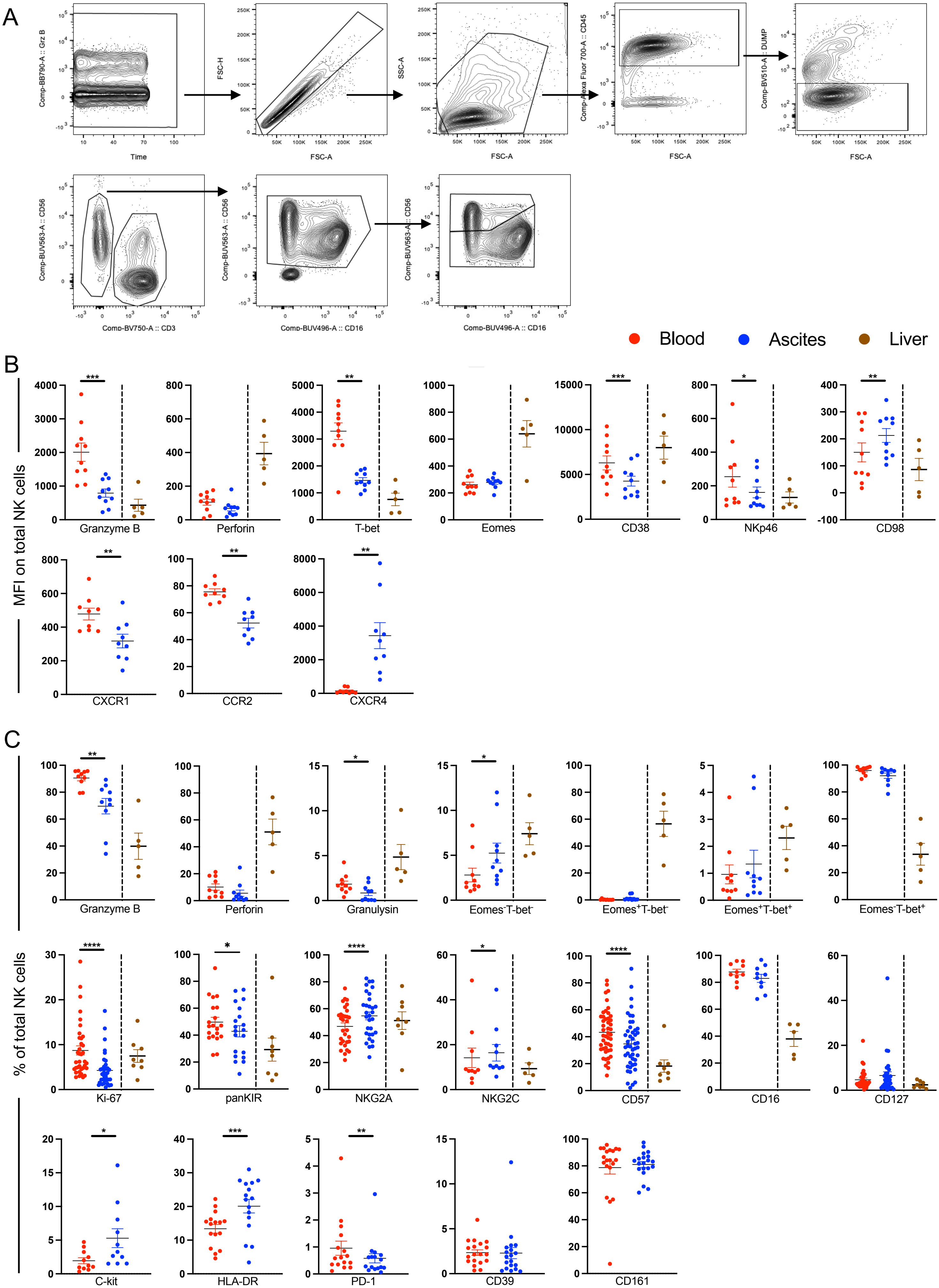

Supplementary Figure 2: UMAP analysis of CD56^{dim} and CD56^{bright} NK cells.

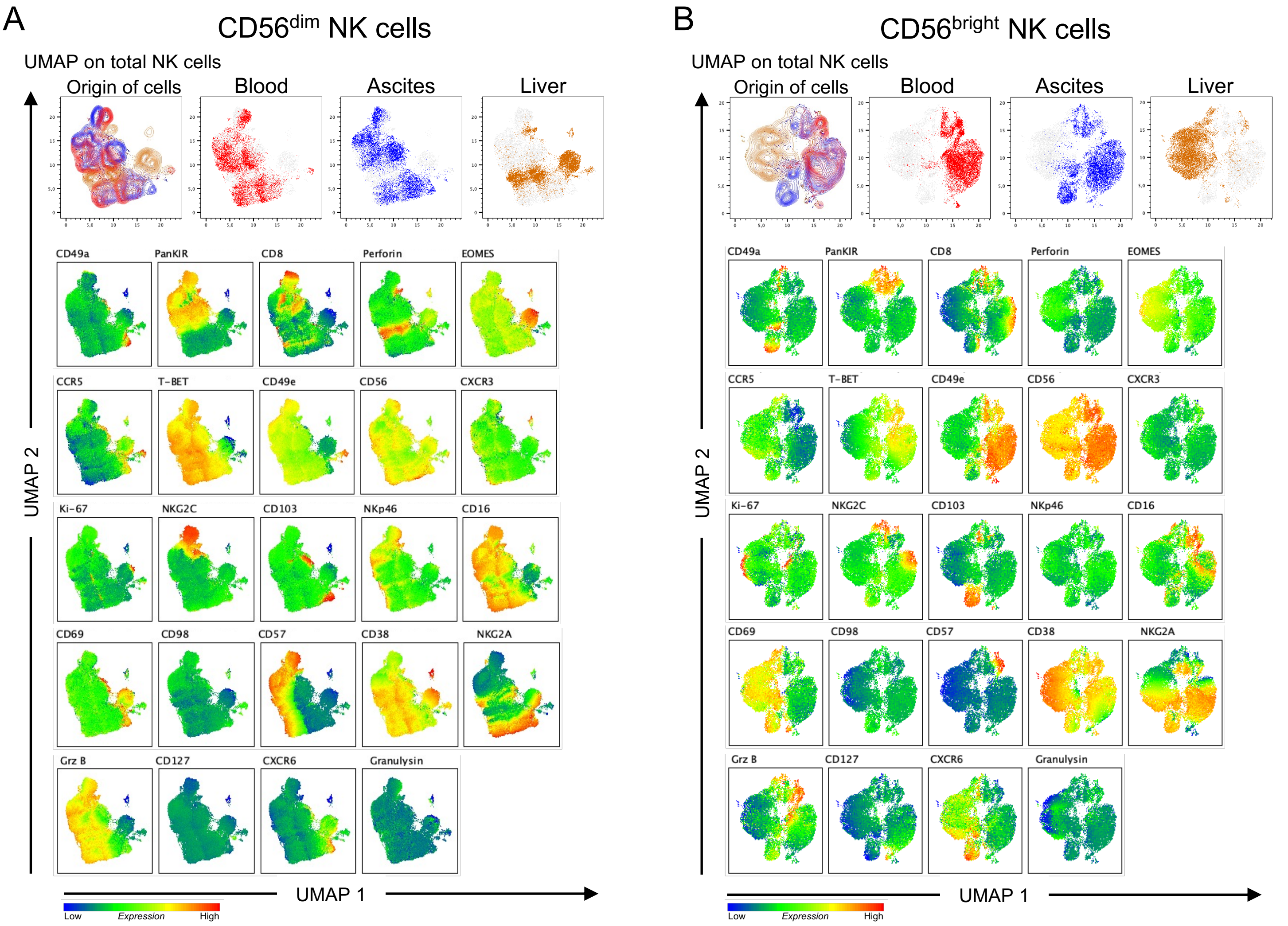

Supplementary Figure 3: Phenotype analysis of CD103$^+$ NK cells and correlation with clinical markers of disease severity.

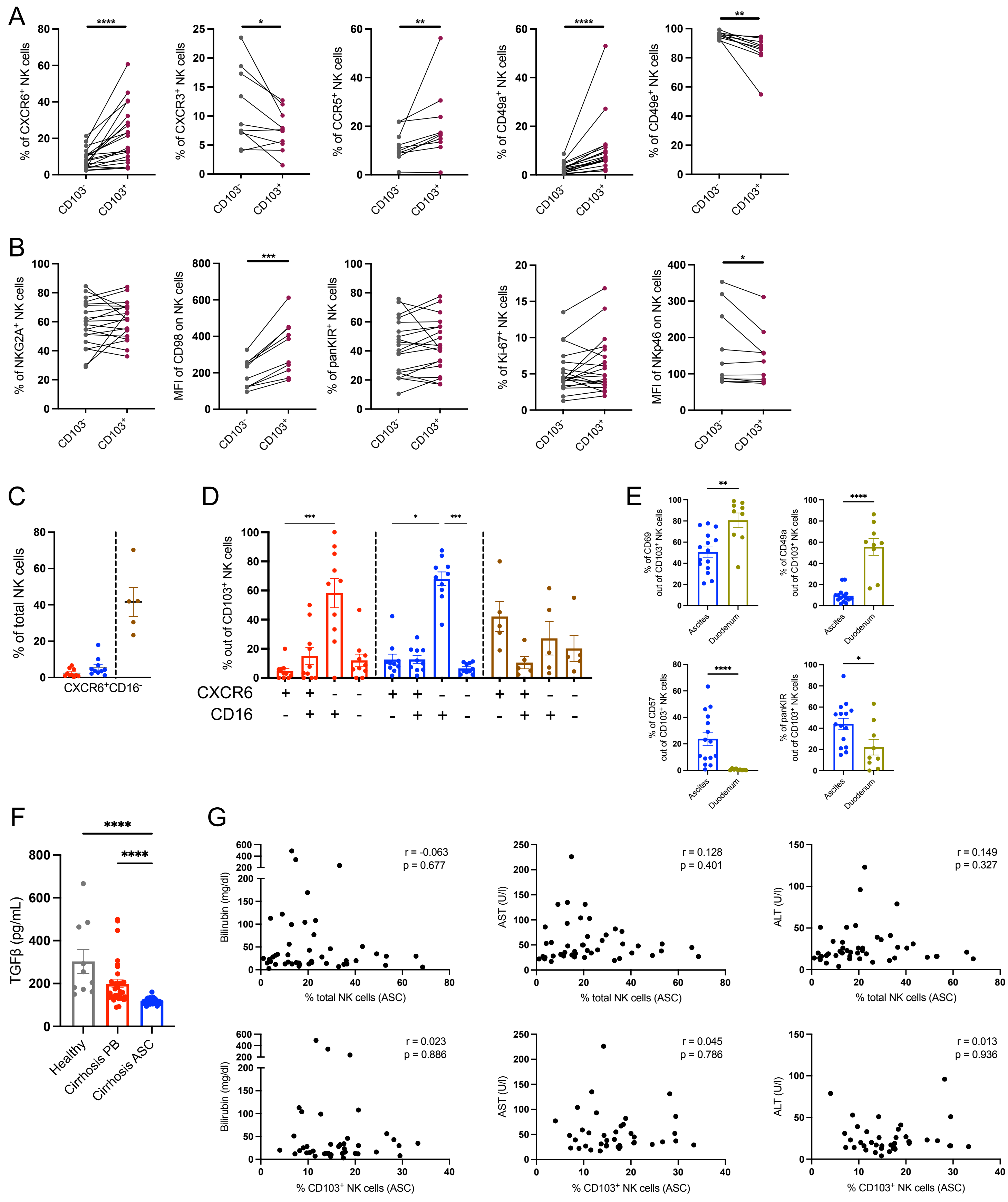

Supplementary Figure 4: Expression of tissue-residency markers on blood NK cells after co-incubation with different agents.

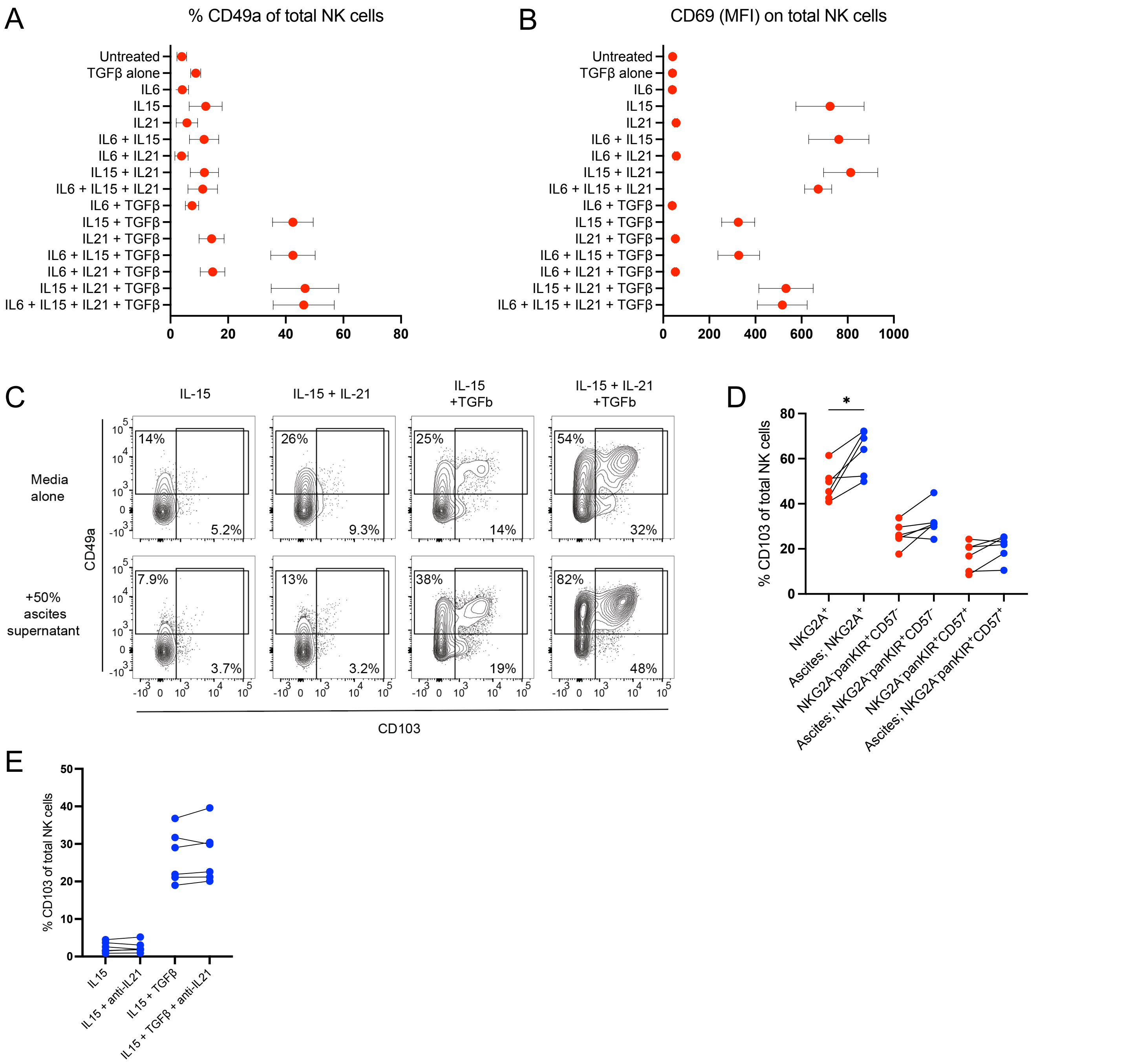

Supplement: Supplementary file 1 — Supporting information [file EJI-55-e51311-s001.pdf]
